# Indents

**Published:** 1921-10

**Authors:** 


### INDENTS

**☞ Brief Articles of Technical or Scientific Value will receive notice and comment. Contributions invited.**

---

Dentist, Know Your Banker. Every man, whether engaged in professional activities or in the affairs of business, owes it to himself to make the acquaintance of his banker. This statement may be taken broadly, since in urban centers where banking institutions are officered by a large number of executives, it might be rather difficult for an ordinary depositor to know them all. However, in an acquaintance with some one of those officials may be readily cultivated and advantageously developed.

In the economic adjustment through which the world is at present passing, the banker holds a unique position. Looking back, we find that the bank, in times past, was looked upon as a sort of financial refrigerator (so cold and dignified was everything about the place, even its officers and clerks). It was a place where money was "kept" and where withdrawals were coldly frowned upon and the withdrawer scrutinized rigidly. The banker was a stern, actually gruff, individual, having an exalted opinion of himself. In the smaller towns he was looked up to as a superior personage in the community one that stood head and shoulders above his townsmen. Now-a-days, however, banks are real human institutions existing for and operated by real human beings for the benefit of their fellow men.

The banker is no longer the power of wealth set apart from others. He has been superseded in that capacity by the manufacturer. Nevertheless the banker and the business man are constantly being drawn more closely together. One is equally dependent upon the other. The bank is becoming known as a service station—a financial service station. Banks are finding that it is to their interest to maintain a friendly and helpful attitude towards all customers, whether their deposits be large or small.

This closer contact between banker and business man is of great assistance in the establishment of confidence—one of the requisites in matters of the inter-change of business and business courtesies. F. W. Taussig, in his book, "Principles of Economics," tells us that "two things are essential for the development of * * * banking; or rather two phases of one thing—confidence." It can be easily seen that to start out the depositor must have confidence in the banker to whom he entrusts his savings, and as the relations continue with the bank, confidence is inspired on the part of the bank and its officers in the probity of the depositor.

The professional man certainly is also entitled to the privileges and courtesies which are offered by the bank as a service station. It is therefore to his advantage to follow the business man's example of cultivating an acquaintance with one or more of the executives of his banking firm. The dentist may at some time desire a loan to assist him in surmounting some unlooked-for obstruction or bridge over some temporary financial gap. This desired help the bank is in a much better position to offer if one or more of its officers has a knowledge of the character and general ability of the dentist who is in need of such assistance. Thus the embarrassing features of such a situation can be greatly lessened.

Then, too, in the event that references are needed for the purpose of opening an account with a supply house, the bank's good will is of great value. A bank reference carries great weight in the establishment of credit relations.

Should the time come when it is advice that is needed, the bank or its officers will be found very helpful owing to its large experience is solving problems of various kinds, and surely a banker will be better able to offer sterling advice in the face of trying conditions when he is possessed of a previous knowledge of his client's occupation, his ability, and his character. Any banker, unless he is grossly dishonest, is humanly interested in the welfare of all the people in his town or community, and therefore can be depended upon to stand ready to aid all those who come to him for advice.

The dentist, no less than the business man, is entitled to the benefits to be derived from this service that is being offered by the banks of today, and he should not fail to take advantage of it.—Carlton Cleveland, in *Dental Facts.*

---

Two Dentists I Know. I know two dentists. Both have added to the wealth I carry about in my mouth.

One of these dentists is an easy-going man. He has a fairly good practice. But I believe most of it has come down through the ages with him.

About six months ago I went to this man for an examination and an estimate on my work. This I got, and made an appointment for a time two weeks later. But when I returned this dentist had forgotten me completely. He didn't even know my name, and it must have been written on his appointment book. He insisted that he had put in several fillings already. He had not. And he was a bit gruff about his questions. His manner was harsh.

But now, let me tell about the other dentist—the crisp-looking man I like. I've told a dozen people about him. I'm a good advertiser for him—but, because he is more than a good dentist. He is a man who knows something about people. He has the human element in him.

I made my first appointment with this dentist by telephone. When I went to fill it I was called by my name. When it was necessary for the office girl to speak to me, again my name was used. It was a courtesy that I liked. It was something, too that didn't just happen.

This dentist was precise in everything that he did. He was well-mannered, knowing just *what* to say and *how* to say it.

He was timely with his pleasant conversation, too. For if my mouth was filled with rolls of cotton and implements he did not bob up with some unnecessary question expecting an answer. But when his work would permit conversation, he made interesting talk—and it was about something else besides weather.

After all, the dentist I like—the man I recommend to my friends—must be a man that I like personally, a man that makes me like him. He must have more than technical ability, he must have nicety of manner. The patients of such a man will prove faithful advertisers, I am sure.—*The Dental Digest.*

---

Cotton Rolls. It has been the practice to place the cotton rolls horizontally, or parallel with the line of occlusion and in the buccal space. But if a stiff cotton roll (large one) about two and one-half to three inches long (the ordinary six-inch roll cut in half) be placed perpendiculary in the mouth, say from the upper first molar to the lower first molar, you have a natural brace, keeping the buccal walls distended, and with the saliva ejector you have a perfectly dry field. Also you have a very effective but gentle means of holding the mouth open.—R. W. Burch.

---

Study of Pulps from Infected Teeth. The authors found active inflammation in fourteen pulps from forty-one infected teeth or 37 per cent—ample confirmation of the opinion previously arrived at from bacteriologic studies that the pulp may be invaded by microorganisms long before it is actually exposed to caries, and may be repeatedly injured long before it actually undergoes necrosis. The research was undertaken to test the validity of the opinion commonly held that an intact wall of dentin protects the pulp from infection, so that only caries or a trauma can make it possible for microorganisms to gain access to the pulp. Connected with this belief is the opinion that pain occurring previous to actual exposure of the pulp must be attributed to the presence either of temperature change or of lactic acid absorbed through the dentinal tubules, these two factors producing pain through the induced hyperemia. The authors have long held the opposed belief that microorganisms can invade the pulp in the absence of caries or before the latter has progressed. The invading organisms, however, do not, in the authors' opinion, set up suppurative or gangrenous processes but instead a granulomatous condition which ends in fibrosis. These alterations naturally pave the way for ordinary infectious processes. In the research to uphold or controvert this belief, the authors made studies of fifty vital teeth and the results which are stated in the opening paragraph of the abstract appear to bear out their views.—Henrici and Hartzell in *Journal of Research.*

---

Dental Caries with General Affections, Especially Gout and Rheumatism. The author's serial article, which closes in this number, terminates with the following conclusions: a marked degree of caries and rheumatism are almost without exception associated together, while between caries and gout there is no demonstrable connection. In pronounced caries the sulphocyanide content of the saliva is notably reduced, and this status is also found in rheumatism and chronic arthritis in general. In typical gout on the other hand the reaction for sulphocyanide is positive. This research of the saliva can give information concerning disturbed metabolism and can also assist in the differentiation of gout from other forms of chronic arthritis. To consider the rheumatism and caries association in more detail the author points out that in nearly 100 per cent of the rheumatic individuals examined caries was so marked as to interfere seriously with mastication. The diminished content of sulphocyanide is at present beyond explanation. In some of the chronic arthritides there is an infected condition of the organism which argues for metabolic disturbances, but on the other hand buccal infection may have originally been responsible for these arthritides. Finally in many cases there is no evidence of infection of any kind, and we are forced to assume the existence of some inborn constitutional inferiority. We can only state that defective sulphocyanide production indicates lowered resistance to disease.—Levy, *International Journal,* July, 1921.

---

INTERSTITIAL GINGIVITIS OR SO-CALLED PYORRHEA ALVEOLARIS: AN INCIPIENT FORM OF SCURVY. In this instructive article the author shows that interstitial gingivitis a disease of the alveolar process and a forerunner of pyorrhea alveolaris, is far more frequent and important than is ordinarily assumed. Only about ten per cent of patients have the advanced pyorrhea stage in which pus is present, whereas everybody sooner or later has interstitial gingivitis in some degree. Being a transitory structure, the alveolar process, after the second dentition, begins to undergo absorption, as the result of the slightest irritation, or in consequence of malnutrition and metabolic changes. The peculiar situation of the alveolar process as an end-organ makes it very susceptible and sensitive to irritations and nutritional influences. Because of the transitory nature of the alveolar process, most systemic changes are first observed in the gums and the alveolar process. The disease would probably be more correctly interpreted if it were called incipient scurvy. A lowered vitality and neglect assist in the production of this disease. What is considered scurvy in many patients is only interstitial gingivitis, or the milder forms of scurvy, in which other bodily symptoms are not present and in which only the gums and alveolar process are involved, owing to their transitory nature. The author's researches have shown that interstitial gingivitis is not an infectious disease. He has shown that the alveolar process is the first structure involved in malnutrition, faulty metabolism, and scurvy-producing interstitial gingivitis. Bone-absorption takes place first; afterwards, inflammation appears. This period is always recognized by bleeding of the gums. Finally, pyorrhea alveolaris may occur. Disorders of the alveolar process should be considered as one of the most important diagnostic signs of faulty metabolism, since the alveolar process has been shown to be the first involved in systemic disturbances due to changes in climate, high altitudes, monotony of diet, and so on. The pathology of interstitial gingivitis, or pyorrhea alveolaris, and of scurvy, is identical, and a uniform term for the disease is therefore to be desired.—E. S. Talbot, *International Journal,* July, 1921.

---

TAKING THE BITE FOR COMPLETE UPPER AND LOWER DENTURES. It is well known how great is the tendency for a patient to push the mandible forward when the bite is being taken, and unless this can be overcome the operation is valueless.

Palpation of the condyles discloses this movement as a slight subluxation of the temporo-maxillary joint, the reality of which is demonstrated by the sound which may be noticed when—the contraction having been overcome—the operator frees the edge of the glenoid cavity from the condyle, and this warns him that his object has been attained. The same false prognathism appears when one asks a patient to close the mouth without any intervention on the part of the operator. It seems that the patient had unlearnt the movement which results in correct closure of the jaws; having no longer any guide to help him in the accomplishment of this movement he appears lost.

Fortunately among the cases which come to us there are many which can give us the secret of their bite; it is for the operator to take advantage of this fact and save himself trouble in the future.

Usually the case presents itself in the following way: The patient requires the extraction of numerous roots and a few loose elongated teeth, or these may be carious, and later on wishes to have full upper and lower dentures. If the mouth is carefully examined before any extraction is performed nine times out of ten one will notice that there persists at least one point where the two jaws come in contact when in natural occlusion. Some times even one tooth or even a root which has an antagonist is sufficient for the patient to use them as a guide in closing the mouth normally.

This circumstance is most valuable, and must be made use of. Proceed to extract the necessary teeth but jealously preserve those teeth or roots which have a point of contact when the jaws are brought together.

Take temporary impressions in composition and the bite in the usual way; then the extractions may be finished. This bite is correct, and if the guide were whole teeth you will at the same time have obtained the height of the denture you are about to make.

Keep this wax bite, which will be of use later, when the impressions for the permanent plates are taken in plaster and you will be able to set up the cases without groping for the exact bite.—Dr. Door, in *La Semaine Dentaire.*

---

Conservatism in Extracting. I agree with those who advise the extraction of dead and diseased teeth after a thorough attempt to effect a cure has failed or is doubtful. It is more than likely that seventy-five per cent of the shadows in the roentgenograms at the apical ends of the teeth have been caused by irritation from mechanical treatment and the use of drugs in dental operations, and hence these teeth are removed unjustifiably. I have seen teeth that Roentgen-ray examination indicated were abcessed—as some men would say—remain fifteen years or longer without any symptom of disease, local or general.—Wm. M. Fine, *A. M. A. Journal.*

